# Nonsteroidal Anti-Inflammatory Drugs for Wounds: Pain Relief or Excessive Scar Formation?

**DOI:** 10.1155/2010/413238

**Published:** 2010-07-04

**Authors:** Wen-Hsiang Su, Ming-Huei Cheng, Wen-Ling Lee, Tsung-Shan Tsou, Wen-Hsun Chang, Chien-Sheng Chen, Peng-Hui Wang

**Affiliations:** ^1^Department of Obstetrics and Gynecology, Yee-Zen General Hospital, Tao-Yuan county 326, Taiwan; ^2^Graduate Institute of Systems Biology and Bioinformatics, National Central University, Jhongli city, Tao-Yuan county 320, Taiwan; ^3^Institute of Statistics, National Central University, Jhongli city, Tao-Yuan county 320, Taiwan; ^4^Department of Obstetrics and Gynecology and Institute of Clinical Medicine, National Yang-Ming University School of Medicine, Taipei 112, Taiwan; ^5^Department of Life Science, National Central University, Jhongli city, Tao-Yuan county 320, Taiwan; ^6^Department of Medicine, Cheng-Hsin General Hospital, Taipei 112, Taiwan; ^7^Department of Nursing, Taipei Veterans General Hospital, Taipei 112, Taiwan; ^8^Department of Obstetrics and Gynecology, Taipei Veterans General Hospital, Taipei 112, and National Yang-Ming University Hospital, Ilan 260, Taiwan

## Abstract

The inflammatory process has direct effects on normal and abnormal wound healing. Hypertrophic scar formation is an aberrant form of wound healing and is an indication of an exaggerated function of fibroblasts and excess accumulation of extracellular matrix during wound healing. Two cytokines—transforming growth factor-*β* (TGF-*β*) and prostaglandin E2 (PGE2)—are lipid mediators of inflammation involving wound healing. Overproduction of TGF-*β* and suppression of PGE2 are found in excessive wound scarring compared with normal wound healing. Nonsteroidal anti-inflammatory drugs (NSAIDs) or their selective cyclooxygenase-2 (COX-2) inhibitors are frequently used as a pain-killer. However, both NSAIDs and COX-2 inhibitors inhibit PGE2 production, which might exacerbate excessive scar formation, especially when used during the later proliferative phase. Therefore, a balance between cytokines and medication in the pathogenesis of wound healing is needed. This report is a literature review pertaining to wound healing and is focused on TGF-*β* and PGE2.

## 1. Introduction

Prostaglandin (PG) E2 (PGE2), synthesized from arachidonic acid by cyclooxygenases (COX) and synthases (PGES), acts as both an inflammatory mediator and fibroblast modulator [1]. The release of PGE2 from skin tissue after toxic stimuli produces local edema and hyperalgesia [2]. PGE2 is the lipid mediator of inflammation in diseases, such as rheumatoid arthritis and osteoarthritis, and is also involved in skin inflammation. Conventionally, nonsteroidal anti-inflammatory drugs (NSAIDs) or their selective cyclooxygenase-2 (COX-2) inhibitors are reported to inhibit PGE2 production and act as effective pain-killers, since they are able to reduce inflammation successfully [3, 4]. In addition, NSAIDs are relatively inexpensive, readily available and familiar; they are often prescribed and used postoperatively for pain control [5]. However, the impact of NSAIDs or COX-2 inhibitors on wound healing is highly controversial, since in theory, an anti-inflammatory agent, like one of the COX-2 inhibitors, may have a negative effect on wound healing. 

The inflammatory process has direct effects on normal and abnormal wound healing. Clinical experience suggests that hypertrophic scar formation is an aberrant form of wound healing [6], involving an exaggerated function of fibroblasts and excess accumulation of extracellular matrix (ECM) during wound healing [7]. 

Although a better understanding of the mechanism of wound healing can be presumed from the increased number of *in vitro* or *in vivo* experiments, and a better treatment algorithm to maintain a regulated and orchestrated inflammatory response will be developed and result in effective and normal wound healing [8–10], most *in vitro* data derived from fibroblasts cultured from keloid lesions only represent the terminal stage of this disease and *in vivo* animal models might not present a real condition in humans.

## 2. The Process of Wound Healing and Skin Inflammation

Compared to Drosophila, similar transcription factor regulates formation and maintenance of the epidermal barrier in mice. These findings suggest that the mechanisms involving wound repair have been conserved by forces of evolution for 700 million years [11]. The secret of wound healing might be hidden in the differences between fetal and adult skin, and why fetal wounds heal without a scar *in utero* [12]. 

**Figure 1:**
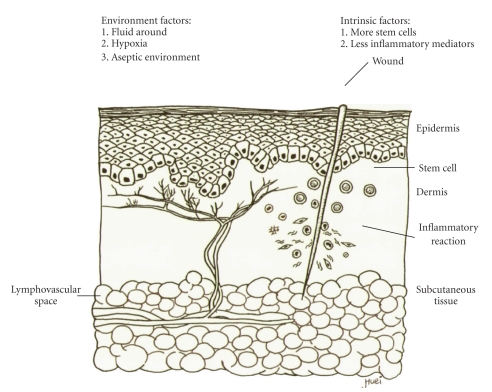
Wound healing of fetal skin with little scarring. Very little inflammatory reaction occurs in fetal skin, which results in little scarring and nearly perfect recovery of fetal skin. Several environment and intrinsic factors are believed to play a role in this process.

**Figure 2:**
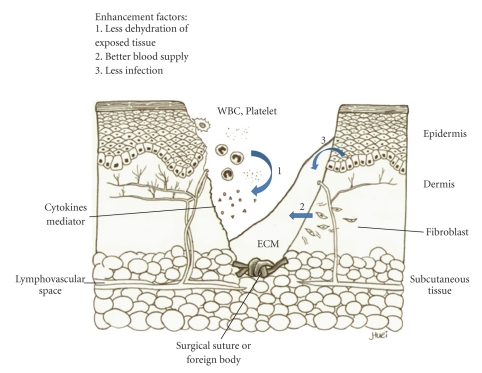
Normal process of wound healing. The initial inflammatory phase begins at the time of wounding, when the activation of the coagulation cascade causes the release of cytokines that stimulate chemotaxis of neutrophils and macrophages into the wound to begin early debridement (1). The proliferative phase is signified by an abundance of fibroblasts and an accumulation of extracellular matrix (ECM) (2). The abundant ECM is then degraded and the immature type III collagen of the early wound is modified into mature type I collagen in final remodeling, or mature phase. A good healing process involves several enhancement factors.

As shown in Figure 1, very little scarring occurs in fetal skin, which results in nearly perfect recovery of fetal skin after trauma. Therefore, understanding the cellular and molecular processes during wound healing is crucial to clarify the pathogenesis of hypertrophic scarring and develop more successful treatment modalities (Figure 2). The known process of normal wound healing involves 3 overlapping phases, inflammation, proliferation, and remodeling. The initial inflammatory phase begins at the time of wounding, when the activation of the coagulation cascade causes the release of cytokines that stimulate chemotaxis of neutrophils and macrophages into the wound to begin early debridement. This will proceed for 2 to 3 days and then the proliferative phase, signified by an abundance of fibroblasts and an accumulation of ECM, fades in and lasts for 3–6 weeks. The follow-up final remodeling, or the mature phase, may take 6–9 months. The abundant ECM is then degraded and the immature type III collagen of the early wound is modified into mature type I collagen [13].

## 3. The Pathogenesis of Excessive Scarring

**Figure 3:**
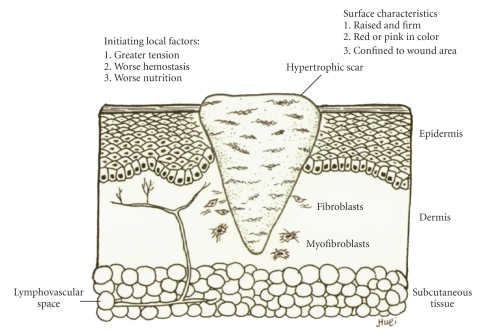
The hypertrophic scarring healing result. A hypertrophic scar possesses several surface characteristics that distinguish it from normal scar formation. There are several initiating factors participate the process. Due to a slow and prolonged regression phase, excessive type III collagens excreted from fibroblasts are accumulated in a direction parallel to epidermal surface. The evolution of myofibroblast from fibroblast may cause wound retraction in the future.

**Figure 4:**
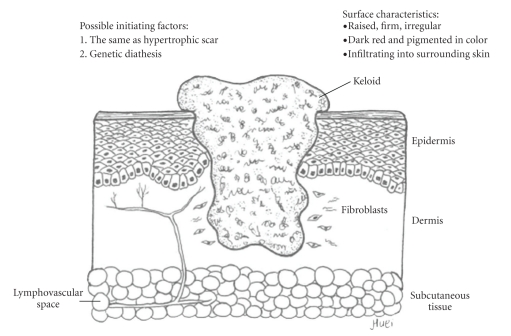
The keloid healing result. Several surface characteristics of keloid mark its difference from hypertrophic scar. However, there is only one difference in the initiating factors. There is no remolding phase in this aberrant wound healing process, and fibroblasts will not eventually turn into myofibroblasts. The abnormal scar is composed of disorganized type I and III collagens with thick irregular branched septa. On the cut surface, tongue-like advancing edges provoke its local advanced infiltrating nature.

Although hypertrophic scarring or keloid formation are regarded as different disease entities based on their patho-histological data (Figures 3 and 4), they still share some common characteristics, including increased fibroblast function, excessive accumulation of ECM, and the common initial inflammatory phase. Keloid fibroblasts (KFs) are supposed phenotypically different from those of hypertrophic scarring, because patients with keloid diathesis do not always form abnormal scars [13]. Either an ambiguous beginning of the inflammatory signal of the inflammatory phase, extending to the proliferative process, or a failure of appropriate degradation and apoptosis may contribute to the pathogenesis of excessive scarring. 

Many local factors are believed to increase the chance of excessive scarring [14, 15]. A hypertrophic scar often results from a wound which was closed in great tension, with rough handling and inadequate hemostasis and debridement, and material with a powerful foreign body reaction, and without enough nutrition. These factors, including transforming growth factor-*β* (TGF-*β*) and PG will be reviewed in the *in vitro *and *in vivo* models subsequently.

## 4. The In Vitro Studies

Hypertrophic scars and keloids represent a dysregulated response to cutaneous injuries, which results in an excessive deposition of collagen. A lot of experiments [16–25], involving keratinocytes, fibroblasts, myofibroblasts, and neutrophils, were designed to test presumed important factors like TGF-*β*1 [16], matrix metalloproteinases (MMPs) [17], and nitric oxide (NO) [18], and to determine whether the hypertrophic process is turned on or how the aberrant apoptosis in the healing course is turned off. Almost all of these studies focused on the aberrant proliferative and remodeling phases. The main reason is not clear, but the possible cause is that the prominence of the aberrant expression of the mediators of wound healing is found during the proliferative and remodeling phases (see below). However, the aberrant wound healing process might occur during a much earlier phase. For example, dysregulated interactions of both epidermal-derived cytokines (interlukin 1: IL-1 *α* and tumor necrotic factor *α*: TNF-*α*) and dermal-derived inflammatory/angiogenic mediators in the beginning of the inflammatory phase contributed to excessive wound scarring [26].

The TGF-*β* family is a key factor in fibrosis, because it is involved in many fibrotic diseases [20, 27]. Upregulation or overexpression of TGF-*β* in keloid keratinocyes [19] and KF [18, 20, 21] results in excessive fibrosis and increases wound scarring. Several treatment protocols, including flash lamp pulsed-dye laser (PDL) [28], exogenous PGE1 [29], PGE2 [1], NO antagonist [18], and TGF-*β* antagonist [30], could ameliorate keloid fibrosis, which is mediated by inhibiting TGF-*β* production in the wound.

Compared with the well-accepted role of TGF-*β* in wound healing, the role of PGE2 is often overlooked, although evidence shows that an elevated PGE2 level is an indicator of progression of inflammation in various kinds of cells and variant inflammatory diseases [31–36]. In addition, a lower level of PGs in fetal skin tissue might indicate a less severe inflammatory reaction, which results in little scar formation after wound healing [12, 37].

PGE2 was shown to decrease fibroblast proliferation, inhibit collagen synthesis, and enhance the expression of MMPs. KF produced less PGE2 than that produced by control fibroblasts [35]. Moreover, the antifibrotic effect of PGE2 during keloid formation was prohibited and could be restored by exogenous PGE2 supplementation [1]. The increased collagen synthesis in KF might be due to decreased PGE2 and cAMP production.

Mechanical compression of the wound may induce excessive scarring and influence the release of PGE2 and the expression of collagenases. PGE2 basal levels in hypertrophic burn scars were significantly lower than those present in normotrophic burn scars [18]. The best prevention and control of hypertrophy, especially in burn scars, is achieved using elastocompression, and compression induced a significant increase in the release of PGE2, in both the remission and active stages, suggesting a role for PGE2 in the process of hypertrophy remission induced by pressure therapy [38].

Another possible problem is the defect in apoptosis and growth during excessive scarring, which hinders the disappearance of both fibroblasts and myofibroblasts at the end of healing [23]. PGs, a promoter of stem or progenitor cell proliferation and tissue regeneration, and positively acting on the downstream of the cascades of apoptosis, may prevent excessive scarring [39]. Table 1 summarizes the different expressions of TGF-*β* and PGE2 in the normal and aberrant process of wound healing.

Besides, PGE2, PGE1, and its analog can increase the activity of collagenase, which is lower in the supernatants from hypertrophic scar fibroblasts culture [40]. PGE1 may have a role in the prevention of hypertrophic scarring by increasing the activity of type I collagenase [29]. However, there is still some controversy regarding the effect of PGs on the process of wound healing. Tranilast, an antiallergic drug inhibiting the release of substances such as histamine and prostaglandins from mast cells, was reported to suppress the collagen synthesis of fibroblasts derived from keloid tissues [22]. It is also believed to suppress collagen synthesis by fibroblasts through inhibiting TGF-*β*1 and PGE2 production and cell proliferation by fibroblasts through inhibiting IL-1 production by inflammatory cells such as macrophages [41]. Lower levels of PGs after tranilast seemed not to result in excessive scarring in this situation.

## 5. The In Vivo Studies

Different animal models like rat [37, 42, 43], mouse [44–46], rabbit [47], lamb, and pig [48, 49] were used to test anti-inflammatory response of TGF-*β* and other mediators of inflammation. However, animal study of specific phase of the wound healing process is almost not possible.

An elevated endogenous TGF-*β* level in animals impedes wound healing and provokes excessive scarring. Elevated TGF-*β* and delayed wound healing in transgenic mice with TGF-*β*1 overexpression were associated with profound inflammation throughout all stages of wound healing and no benefit in wound healing [45]. Injection of an antibody of TGF-*β* in a rat demonstrated that inhibition of TGF-*β* reduced scar formation in adult wound healing [37]. Topical application of a synthetic TGF-*β* antagonist accelerated re-epithelialization in pig burn wounds. It also reduced wound contraction and scarring in standard pig skin burns, pig skin excision, and rabbit skin excision wounds [47, 49]. 

An initial inflammatory response with elevated PGE2 can be effectively blocked by medicine or even dietary fat in animal models. A toxin-induced inflammatory response characterized by an early exudative phase is accompanied by PGE2 production, and a late proliferative phase associated with COX-2 induction is effectively inhibited by COX-2 inhibitors in rats [43]. Modification of dietary fat intake might inhibit fever via a reduced release of PG, probably within the brain, but does not affect the local or afferent signals involved in fever generation [50].

Mechanical stress in the proliferative phase was necessary to replicate hypertrophic scar formation in a mouse model of hypertrophic scarring. It is believed that mechanical loading early in the proliferative phase of wound healing produces hypertrophic scars by inhibiting cellular apoptosis [46]. However, an animal that does not form keloid growth like a human does cannot be an ideal model for research.

## 6. The Various Modalities of Treatment

As reported in the studies mentioned above, many treatment modalities for excessive scarring have been proposed, but no complete and satisfying remission has been achieved. The treatment modalities include surgical excision, radiation, corticosteroid injections, cryotherapy, laser vaporization, topical 5-fluorouracil [51], bleomycin injection [52], paper tape to eliminate scar tension [53], pressure garment therapy [54], silicone gel sheeting [55], and short-term use of ozonated oil [56, 57]. Since we still lack an in-depth understanding of the underlying mechanism responsible for excessive inflammation and scarring, no single modality has shown an absolute, complete cure rate. At present, the multimodality approach to scarring control has shown significant benefits [56–58]. The most effective of the scar-reducing protocols likely entails a polytherapeutic strategy for management. Further investigation into the role of inflammation in scarring is paramount to the development of improved scar-reducing agents. There is a need for large controlled trials using a polytherapeutic strategy that combines existing and novel agents to provide a standardized evidence-based evaluation of efficacy [9].

A rising number of novel therapeutic agents, like TGF-*β* antagonists [47, 49], exogenous PGE2 [1], and stem cell therapy [59], are currently under development, encouraged by emerging preliminary findings in both animal models and human studies. The hypertrophic scar/keloid treatment algorithms that are currently available are likely to be significantly improved in the future by high-quality clinical trials.

## 7. The Study Models

A detailed quantitative model of the wound healing process, including re-epithelialization, epidermal differentiation, cell migration, proliferation, inflammatory response, dermal closure, matrix distribution, and skin remodeling, may be utilized as a diagnostic platform for standardizing the assessment of wound healing progression, as well as a screening tool for potential therapies [60].

With advances in biomolecular techniques, high-throughput study tools make a genomic or proteomic scale study of the inflammatory cascade become possible [61], and a biochemical model or so-called inflammatomics study will aid in understanding the overall picture of the healing process after injury.

On the other hand, a more ideal *in vivo* model, other than an animal model, is required for clinical studies. The group of patients suffering from excessive scarring after Cesarean section seems a good model, because it is the most frequent and common surgical procedure in reproductive-age women. In addition, these women have an opportunity to remove the hypertrophic scar or keloid lesion, since many become pregnant again and schedule an elective repeated Cesarean section. Therefore, a useful treatment modality to prevent the recurrence of hypertrophic scarring after scar removal is needed and worthy of research. In fact, there are some reports on the reduced scar formation after an improvement has been made in surgical techniques [62, 63].

## 8. Conclusion

Better understanding of the pathogenesis of wound healing will eventually contribute to progress in the treatment of excessive scarring. Whether excessive scarring occurs or not might be decided at the moment when the first inflammation response is initiated once the wound is established. PGEs might play a role in the prevention of excessive scarring. A thorough study and understanding of the inflammatory cascade will help us cope with a lot of diseases, including hypertrophic scarring and keloid.

## Figures and Tables

**Table 1:** The levels of PGE2 and TGF-beta during the normal and aberrant wound healing process.

	Normal Healing Process			Excessive Scarring		
Phases	1*	2	3	1	2	3
TGF-beta	↑	↑	↓	↑	↑↑↑	↑↑
PGE2	↑	↑	↑	↑	↓↓↓	↓↓
Net Effect	debrided	limited	mature	unknown	excessive	delayed

*1 for inflammatory phase, 2 for proliferative phase, and 3 for remodeling phase.
